# Disability from cardiovascular diseases at Osh city, Kyrgyz Republic

**DOI:** 10.6026/973206300200110

**Published:** 2024-02-29

**Authors:** Tazhidinovich Gulamov Isabek, Kalmatovich Kalmatov Romanbek, Sagynbaevich Sydykov Akylbek, Temirzhanovich Abylov Kuttubek, Nurlan Raiimbekuulu, Akbalaevna Akbalaeva Begimai, Siddiqui Mohd Faizan

**Affiliations:** 1 International Medical Faculty, Osh State University, Osh City, Kyrgyzstan; 2 Universität Gießen Fachbereich Medizin, Giessen, Germany

**Keywords:** Cardiovascular disease, disability indicators, LHA, medical efficacy, rural-urban disparities

## Abstract

Cardiovascular diseases (CVDs) are the leading global cause of death, contributing to health deterioration and increased healthcare expenses. Therefore, it is of interest to investigate the disability rates related to cardiovascular diseases at Osh city, Kyrgyz Republic. We report the prevalence of disability in both urban and rural areas, highlighting the impact of regional disparities in medical and social services. Data shows that adult cardiovascular disease impairment in Kyrgyzstan suggests challenges in accessing medical and social support, particularly in rural regions. Thus, the rural-urban divide in critical disability metrics impedes equitable research. Comprehensive assessments and interventions are imperative to mitigate cardiovascular diseases and associated disabilities in both rural and urban populations at Kyrgyz Republic.

## Background:

The medical and social significance of cardiovascular diseases (CVD) lies not only in the high rates of morbidity and mortality from this pathology but also in the high level of disability among patients [1,2, 3]. In recent years, CVD, mainly ischemic heart disease and arterial hypertension, have been the leading causes of disability in the Russian Federation and several other countries [4- 5]. Beyond the negative impact on the population's health profile, the disability of patients inflicts serious economic damage. This includes costs associated with social support for disabled individuals and their families, as well as the unemployment of the disabled. It is known that the disability of individuals of working age significantly reduces the labour potential of the state [6-7]. Investigating the dynamics of disability indicators and structural alterations resulting from circulatory system diseases facilitates the discernment of patterns in their manifestation, with due consideration given to regional variations [8-9]. While it remains imperative to grasp the fundamental physiological determinants underlying mortality, conducting comprehensive evaluations of the principal factors contributing to diseases presents additional avenues to elucidate public policy frameworks. In the Kyrgyz Republic (KR), the UN Convention on the Rights of Persons with Disabilities was ratified in 2019, making the study of various aspects of disability among persons with limited health abilities (LHA) in this territory a current issue [10- 11]. Therefore, it is of interest to evaluate how common disability caused by cardiovascular disease (CVD) is among the population of the Osh region in the Kyrgyz Republic from 2016 to 2021.

## Methodology:

The research undertaken in this study was extensive, spanning the years 2016 to 2021. The study included a detailed examination of individuals with limited health abilities (LHA) under medical supervision in healthcare institutions, as well as primary disability rates attributed to cardiovascular diseases (CVD) in both the adult and paediatric (up to 18 years) populations of the Kyrgyz Republic (KR), with a particular focus on the city of Osh and the Alai and Chon-Alai districts of the Osh region. The major sources of information used were district medical-social expert commission reports and minutes of meetings. The study included a mix of statistical, analytical, and epidemiological methods. All statistical analysis was conducted on SPSS version 23.

## Results:

Disability due to diseases of the circulatory system (DCS) from 2016 to 2021 consistently held the leading position in the structure of disability among the adult population of the KR. During this period, there was a steady increase in the number of LHAs from 14,935 cases (38.9 per 10,000 populations) to 16,387 cases (39.4 per 10,000 populations) (Table 1). Compared to the city of Osh, the number of Persons with Limited Health Abilities (LHA) in the Osh region from 2016 to 2021 was significantly lowers (Table 2). In the structure of disability among the child population, disability due to diseases of the circulatory system (DCS) is only in 14th place. However, similar to adults, there is a trend of increasing numbers of Persons with Limited Health Abilities (LHA) under 18 years of age, from 247 cases (1.1 per 10,000 inhabitants) in 2016 to 294 cases (1.2 per 10,000 inhabitants) in 2021 (Table 3). In 2016, among the adult population of the Kyrgyz Republic (KR), 7,530 residents were initially recognized as disabled. By 2021, this number increased to 7,869 residents. Throughout the years of observation, the majority of those newly recognized as disabled were individuals with diseases of the circulatory system (DCS): 1,422 in 2016; 1,474 in 2017; 1,461 in 2018; 1,488 in 2019; 1,205 in 2020; and 1,494 in 2021 (Table 4).

A comparative analysis of persons initially recognized as disabled among urban and rural populations showed that the number of LHAs in the Osh region in 2016 was lower than in the city of Osh (2.4 and 1.7 per 10,000, respectively). In 2017-2019, the numbers were higher; in 2020, the figures were comparable; and in 2021, they were lower again (1.6 and 3.3 cases per 10,000 inhabitants, respectively) (Table 5). Over all the years of observation, the rates of primary disability in the Osh region were lower than in the KR. The number of children initially recognized as disabled in the KR during the period 2016-2021 remained stable, amounting to 0.1 person per 10,000 population. The rates of primary disability among the rural and urban child populations were similar.

## Discussion:

Despite advances in the diagnosis and treatment of cardiovascular diseases (CVD), disability due to diseases of the circulatory system (DCS) remains at a high level in the Kyrgyz Republic (KR) and does not show a decreasing trend [12, 13,14]. In our study, the number of Persons with Limited Health Abilities (LHA) in the Osh region from 2016 to 2021 was lower than in the city of Osh and in the KR as a whole. This trend may indicate less accessibility of medical-social expertise services for residents of remote areas and insufficient accounting of persons with disabilities [15,16,17, 18]. It might be necessary to revise and optimize the operation of medical organizations in rural areas, especially in directing CVD patients who are eligible for disability determination to medical-social examination. [19,20,21]. Rates of primary disability are an important medical-social criterion of public health and reflect the accessibility and effectiveness of regional medical-social programs [22,23, 24]. In our study, the rates of primary disability in the population of the Osh region over the entire observation period were lower than in the KR [24,25, 26,27,28]. However, compared to the urban population, they showed unstable dynamics, complicating their objective assessment. Data from elsewhere [29,30,31, 32] revealed a significant difference in the level of primary disability of the adult and child populations per 10,000 inhabitants for the period 2017-2019 when analyzing the main indicators of the Osh city medical-social expert commission's activities [33,34,35, 36]. According to the authors, this circumstance may be due to the lack of clear criteria for medical-social expertise in this region.

It is known that the majority of disabilities due to cardiovascular diseases (CVD) are found in people over 60 years of age [37,38,39, 40]. Considering the global trend of an aging population, an increase in the number of Persons with Limited Health Abilities (LHA) can be expected in the coming years. For example, according to Guzman-Castillo M. *et al.* (2017), in England and Wales, there is an anticipated 19.4% increase in the population aged 65 and older by 2025, with the number of disabled individuals expected to increase by 25.0% [41-42]. In our study, the level of disability due to CVD among children under 18 remained low throughout the observation period, with leading causes of disability being nervous system diseases, congenital anomalies, mental disorders, and behavioural disorders. However, a population cohort study conducted in Sweden in 2020 found a direct correlation between overweight/obesity and low cardiorespiratory fitness in adolescence and an increased risk of disability due to CVD in adulthood [43]. Therefore, preventive actions in young age, such as strengthening the cardiorespiratory system and maintaining a healthy body weight, can be considered as way to prevent future CVD-related disabilities. Although there are comparatively few children disabled due to CVD, they require close attention from pediatric services, especially in remote rural areas. As noted by Uzakbaev *et al.* (2018) [44], children with limited health abilities in the KR currently face a number of medical-social problems, such as insufficient availability of rehabilitation services, lack of a well-organized system for overcoming environmental barriers by disabled persons, and insufficient effectiveness of psychological-medical-pedagogical consultations, which are entirely absent in remote rural areas.

## Conclusion:

Diseases of the circulatory system (DCS) remain a significant issue in the Kyrgyz Republic (KR) despite progress in identifying and treating cardiovascular diseases (CVD). Data shows that cardiovascular disease-related impairment is prevalent among adults in Kyrgyzstan, especially in rural areas, posing challenges in accessing medical and social support services. This rural-urban gap in disability indicators hinders efforts to achieve research equity. Global projections underscore the escalating burden of cardiovascular disease (CVD)-associated disability, indicating the need for preventive strategies, early interventions during adolescence, targeted legislative reforms, and enhanced healthcare services in Kyrgyzstan.

## Data availability:

The raw data supporting the conclusions of this article will be made available by the authors, without undue reservation.

## Funding statement:

The authors received no financial support for the research, authorship, or publication of this article.

## Figures and Tables

**Table 1:** Number of persons with Limited Health Abilities (LHA)

**Class of diseases**	**Number of Persons with LHA**											
	**Absolute number**						**Per 10,000 population**					
	**Observation years**											
	**2016**	**2017**	**2018**	**2019**	**2020**	**2021**	**2016**	**2017**	**2018**	**2019**	**2020**	**2021**
Circulatory System Diseases	14935	15340	15858	16274	16187	16387	38,9	39,2	40,6	40,3	39,5	39,4
Mental and Behavioral Disorders	13917	14182	14304	14845	14784	14996	36,2	36,3	36,6	36,8	36,1	36,1
Nervous System Diseases	12231	12448	12443	12759	12874	13241	31,8	31,8	31,8	31,6	31,4	31,8
Diseases of the Eye and Adnexa	9821	9791	9637	9712	9678	9436	25,6	25,0	24,6	24,1	23,6	22,7
Musculoskeletal System and Connective Tissue Diseases	6509	6765	7143	7418	7460	7631	16,9	17,3	18,3	18,4	18,2	18,4
Injuries and Poisonings	6306	6449	6574	6742	6851	6907	16,4	16,5	16,8	16,7	16,7	16,6
Endocrine System Diseases, Nutritional Disorders	5303	5585	5902	6313	6427	6667	13,8	14,3	15,1	15,6	15,7	16,0
Neoplasms	4647	4646	4736	4919	4945	5164	12,1	11,9	12,1	12,2	12,1	12,4
Respiratory System Diseases	3764	3845	3991	3983	3965	4043	9,8	9,8	10,2	9,9	9,7	9,7
Certain Infectious and Parasitic Diseases	3319	3282	3300	3396	3365	3306	8,6	8,4	8,4	8,4	8,2	7,9
Congenital Anomalies (Developmental Defects)	3103	3166	3334	3561	3691	3773	8,1	8,1	8,5	8,8	9,0	9,1
Diseases of the Ear and Mastoid Process	2888	2899	2812	2816	2804	2827	7,5	7,4	7,2	7,0	6,8	6,8
Genitourinary System Diseases	2262	2433	2504	2635	2672	2819	5,9	6,2	6,4	6,5	6,5	6,8
Digestive System Diseases	2066	2117	2220	2213	2240	2306	5,4	5,4	5,7	5,5	5,5	5,5
Blood Diseases, Blood-Forming Organs, Immune Mechanism Disorders	445	444	456	456	472	476	1,2	1,1	1,2	1,1	1,2	1,1
Skin and Subcutaneous Tissue Diseases	224	233	230	238	279	288	0,6	0,6	0,6	0,6	0,7	0,7
Total	93193	95177	97070	99679	100183	101902	242,6	243,4	248,2	246,9	244,6	245,0
Note: LHA refers to Persons with Limited Health Abilities, and KR stands for the Kyrgyz Republic.

**Table 2:** Number of persons with Limited Health Abilities (LHA) (Adults) due to Cardiovascular Diseases (CVD)

	**KR**		**Osh city**		**Osh region**	
**Observation years**	**Absolute number**	**Per 10,000 population**	**Absolute number**	**Per 10,000 population**	**Absolute number**	**Per 10,000 population**
2016	14935	38,9	726	39,2	1908	25,0
2017	15340	39,2	715	37,8	1999	25,6
2018	15858	40,6	686	35,7	2078	26,1
2019	16274	40,3	674	34,4	2106	26,0
2020	16187	39,5	664	33,6	2081	25,3
2021	16387	39,4	626	31,2	2103	25,0
Note: LHA refers to Persons with Limited Health Abilities, and KR stands for the Kyrgyz Republic.

**Table 3:** Number of children under 18 years of age with Limited Health Abilities (LHA)

	**Number of Persons LHA, Children (0-17 Years 11 Months 29 Days)**											
**Class of diseases**	**Absolute number**						**Per 10,000 population**					
	**2016**	**2017**	**2018**	**2019**	**2020**	**2021**	**2016**	**2017**	**2018**	**2019**	**2020**	**2021**
Nervous System Diseases	7750	8088	8405	8768	8845	8920	34,6	35,4	36,7	36,2	35,6	35,2
Congenital Anomalies (Developmental Defects)	6015	6348	6753	7175	7375	7418	26,9	27,7	29,5	29,7	29,7	29,3
Mental and Behavioral Disorders	2767	2822	2950	3138	3296	3370	12,4	12,3	12,9	13,0	13,3	13,3
Diseases of the Eye and Adnexa	1525	1553	1539	1546	1534	1459	6,8	6,8	6,7	6,4	6,2	5,8
Musculoskeletal System and Connective Tissue Diseases	1163	1226	1221	1187	1200	1163	5,2	5,4	5,3	4,9	4,8	4,6
Diseases of the Ear and Mastoid Process	935	966	996	1006	1009	1009	4,2	4,2	4,4	4,2	4,1	4,0
Injuries and Poisonings	749	753	807	900	948	907	3,3	3,3	3,5	3,7	3,8	3,6
Genitourinary System Diseases	457	488	506	570	552	574	2,0	2,1	2,2	2,4	2,2	2,3
Endocrine System Diseases, Nutritional Disorders	439	477	518	583	635	733	2,0	2,1	2,3	2,4	2,6	2,9
Respiratory System Diseases	383	413	412	442	444	458	1,7	1,8	1,8	1,8	1,8	1,8
Blood Diseases, Blood-Forming Organs, Immune Mechanism Disorders	369	367	392	390	373	382	1,6	1,6	1,7	1,6	1,5	1,5
Certain Infectious and Parasitic Diseases	309	273	285	274	258	267	1,4	1,2	1,2	1,1	1,0	1,1
Neoplasms	226	267	245	301	312	370	1,0	1,2	1,1	1,2	1,3	1,5
Circulatory System Diseases	247	222	222	249	256	294	1,1	1,0	1,0	1,0	1,0	1,2
Digestive System Diseases	118	150	174	173	166	197	0,5	0,7	0,8	0,7	0,7	0,8
Skin and Subcutaneous Tissue Diseases	67	94	105	108	130	120	0,3	0,4	0,5	0,4	0,5	0,5
Total	24107	25023	26116	27312	27864	28184	107,7	109,4	114,1	112,9	112,1	111,2
Note: LHA refers to Persons with Limited Health Abilities, and KR stands for the Kyrgyz Republic.

**Table 4:** Number of Persons with Limited Health Abilities (LHA) initially recognized at Kyrgyz Republic (KR)

	**Initially recognized as persons with LHA**											
	**Absolute number**						**Per 10,000 population**					
Class of diseases	2016	2017	2018	2019	2020	2021	2016	2017	2018	2019	2020	2021
Certain Infectious and Parasitic Diseases	307	246	283	282	233	238	0,8	0,6	0,7	0,7	0,6	0,6
Neoplasms	707	702	737	728	664	780	1,8	1,8	1,9	1,8	1,6	1,9
Blood Diseases, Blood-Forming Organs, Immune Mechanism Disorders	40	39	36	38	34	40	0,1	0,1	0,1	0,1	0,1	0,1
Endocrine System Diseases, Nutritional Disorders	601	627	639	706	666	779	1,6	1,6	1,6	1,7	1,6	1,9
Mental and Behavioral Disorders	676	540	502	1059	481	695	1,8	1,4	1,3	2,6	1,2	1,7
Nervous System Diseases	882	880	810	772	665	856	2,3	2,3	2,0	1,9	1,6	2,1
Diseases of the Eye and Adnexa	523	558	467	453	358	425	1,4	1,4	1,2	1,1	0,9	1,0
Diseases of the Ear and Mastoid Process	102	92	94	76	76	91	0,3	0,2	0,2	0,2	0,2	0,2
Circulatory System Diseases	1422	1474	1461	1488	1205	1494	3,7	3,8	3,7	3,7	2,9	3,6
Respiratory System Diseases	255	244	240	209	219	268	0,7	0,6	0,6	0,5	0,5	0,6
Digestive System Diseases	208	238	235	235	210	247	0,5	0,6	0,6	0,6	0,5	0,6
Skin and Subcutaneous Tissue Diseases	13	13	19	40	31	24	0,03	0,03	0,0	0,1	0,1	0,1
Musculoskeletal System and Connective Tissue Diseases	516	542	540	602	480	696	1,3	1,4	1,4	1,5	1,2	1,7
Genitourinary System Diseases	256	237	210	259	208	234	0,7	0,6	0,5	0,6	0,5	0,6
Congenital Anomalies (Developmental Defects)	196	189	200	226	173	196	0,5	0,5	0,5	0,6	0,4	0,5
Injuries and Poisonings	639	663	575	525	461	598	1,7	1,7	1,4	1,3	1,1	1,4
Total	7530	7514	7267	7849	6303	7869	19,6	19,2	18,3	19,4	15,4	18,9
Note: LHA refers to Persons with Limited Health Abilities, and KR stands for the Kyrgyz Republic.

**Table 5:** Number of persons with Limited Health Abilities (LHA)

**Observation years**	**KR**		**Osh city**		**Osh region**	
	**Absolute number**	**per 10,000 population**	**Absolute number**	**Per 10,000 population**	**Absolute number**	**Per 10,000 population**
2016	1422	3,7	32	1,7	184	2,4
2017	1474	3,8	67	3,5	162	2,1
2018	1461	3,7	59	3,1	173	2,2
2019	1488	3,7	63	3,2	146	1,8
2020	1205	2,9	32	1,6	148	1,8
2021	1494	3,6	66	3,3	132	1,6
Note: LHA refers to Persons with Limited Health Abilities, and KR stands for the Kyrgyz Republic.
